# Broadband Noise Acoustic Stapedial Reflex Thresholds as a Marker of Cochlear and Neural Integrity in Individuals with Normal Audiograms

**DOI:** 10.3390/audiolres16040105

**Published:** 2026-07-26

**Authors:** Yongqing Liang, Navid Shahnaz, Valter Ciocca

**Affiliations:** 1 Department of Psychology, Zoology, and Cellular & Physiological Sciences, Faculty of Science, University of British Columbia, Vancouver, BC V6T 1Z4, Canada; yliang40@student.ubc.ca; 2 School of Audiology & Speech Sciences, Faculty of Medicine, University of British Columbia, Vancouver, BC V6T 1Z4, Canada; vciocca@audiospeech.ubc.ca

**Keywords:** acoustic stapedial reflex threshold, broadband noise (BBN), BBN advantage, normal audiogram, auditory complaints, speech-in-noise difficulty, cochlear synaptopathy, hidden hearing loss

## Abstract

**Background/Objectives:** Some individuals present with difficulty understanding speech in noise, tinnitus, or both, despite normal conventional audiometric hearing thresholds. This study evaluated whether acoustic stapedial reflex thresholds (ASRTs) could serve as a clinically useful indicator of auditory complaints not captured by conventional audiometry. **Methods:** ASRTs for pure tones and broadband noise (BBN) were measured in 146 ears (73 participants) from controls, music students, and symptomatic participants. Bayesian random-intercept linear regression models were used to compare ASRTs among groups, adjusting for age and sex. ASRT difference scores were also used for ROC analysis and to estimate the difference between pure-tone and BBN thresholds (“BBN advantage”). **Results:** Symptomatic ears showed higher ASRTs than control ears with BBN stimuli, after adjustment for age and sex. Symptomatic ears had a lower AvgDiff posterior mean (reduced BBN advantage) than control ears. ASRTs for music students were directionally consistent with an intermediate pattern, but with wider credible intervals and greater uncertainty. Exploratory ROC analysis showed moderate separation between symptomatic and non-symptomatic ears with AvgDiff; however, symptom status was not independently validated against any diagnostic reference standard. **Conclusions:** The study suggests that a reduced BBN advantage may be associated with auditory complaints in individuals with normal hearing at conventional audiometric frequencies. However, these findings should be interpreted with caution and not used for specific diagnostic purposes, as the symptomatic group was substantially older and symptom status was not independently validated against any diagnostic reference standard.

## 1. Introduction

Hearing loss currently affects 18 percent of the population worldwide [1]. Cochlear synaptopathy, commonly referred to as hidden hearing loss (HHL), is less common, affecting approximately 1 to 10 percent of patients in hearing clinics. It results from damage to the synapses connecting inner hair cells to the auditory nerve [2]. In audiology, HHL is defined as difficulty processing auditory information despite a normal audiogram [2,3]. The literature has proposed that hidden hearing loss may be a clinical indicator for predicting later-life hearing loss; however, this remains debated, as evidence is mixed [2]. HHL has important implications, as hearing difficulties affect daily communication and cognitive function, and early management of hidden hearing loss may reduce the incidence of neurodegenerative disorders such as dementia [2,3,4]. However, there are currently no physiological or behavioral diagnostic markers for this condition in humans. For the purposes of this study, “clinically hidden auditory complaints” refers to patient-reported auditory difficulties despite normal conventional audiometric thresholds, without implying a confirmed diagnosis of cochlear synaptopathy.

Evidence from animal studies suggests that hidden hearing loss is likely caused by permanent damage to synapses between inner hair cells and afferent nerve fibers in the cochlea. Multiple factors, including noise exposure, drugs, and aging, can cause damage to synapses [2,3,5,6]. Although the synaptic loss mechanism has only been confirmed in rodent studies, the prior literature suggests that damage in humans is more likely to occur at synapses connected to nerve fibers with a low spontaneous spike rate (low-SR). Conventional audiometric tests primarily assess high-spontaneous-rate (high-SR) fibers. For this reason, patients with hidden hearing loss typically present with normal audiograms despite underlying neural deficits [2,7]. Taken together, the previous literature suggests that HHL is driven by synaptic dysfunction that is not detectable on standard audiograms, highlighting the need to identify a new diagnostic marker for HHL [2,3,5,6,7].

Recent studies have proposed that in both noise-exposed and aging populations, the synapses between inner hair cells and afferent nerve fibers are more vulnerable than hair cells, supporting the claim that hidden hearing loss may precede overt hearing loss, which is associated with hair cell damage and threshold elevation [6]. In mouse studies, both prolonged and short-term noise exposure have been shown to cause permanent synaptic damage, consistent with the current synaptic loss hypothesis in hidden hearing loss [6,8,9]. However, in human studies, the results remain inconsistent [8,10]. Studies have suggested that there is a non-significant association between noise exposure and HHL in humans, and that lifelong exposure to noise was used to measure human noise exposure rather than short-term noise exposure measures in rodents [10,11]. Hence, this may suggest that aging adults with more lifelong exposure to loud noise are more vulnerable to synaptic damage than younger individuals [12].

Noise-induced hidden hearing loss may also reduce the strength of the acoustic stapedius reflex, a middle-ear muscle reflex. The acoustic stapedius reflex is a protective mechanism that involves the involuntary contraction of the stapedius muscles to reduce the magnitude of prolonged, moderate-to-high-intensity auditory signals transmitted to the inner ear [13,14]. Clinically, acoustic stapedius reflex thresholds (ASRTs) are commonly used as part of the immittance test battery to support the differential diagnosis of conductive, cochlear, retrocochlear, and facial nerve pathway disorders [15]. The average ASRTs for pure-tone stimuli are approximately 20 dB higher than those for broadband stimuli in normal-hearing participants [16]. This difference is known as the acoustic reflex noise-tone difference (“NTD”) or the broadband acoustic reflex advantage (“BBN advantage”) [16,17].

The past literature has reported that in older populations with hearing loss, both pure-tone and broadband (BBN) ASRTs increase in proportion to the degree of hearing loss, with BBN ASRTs rising more steeply. The BBN advantage is calculated as the difference between the average pure-tone and broadband ASRTs. The BBN advantage is reduced in conventional sensorineural hearing loss [18,19]. In summary, research from the 1980s has examined the relationship between hearing loss and the loss of the BBN advantage [18,19,20]. Prior studies provide biological plausibility for examining acoustic reflex measures in relation to subtle auditory dysfunction. Animal studies suggest that cochlear neuropathy can weaken the middle-ear muscle reflex, and recent reviews have proposed acoustic stapedial reflex measures as candidate tools for investigating noise-induced cochlear damage [15,21]. However, human evidence remains inconsistent, and studies of normal-hearing adults have not consistently demonstrated relationships between acoustic reflex thresholds, tinnitus, speech-in-noise perception, and noise exposure [12,15,21,22]. Thus, the present findings should be viewed as exploratory and require validation against independent physiologic measures.

The main purpose of this exploratory study was to determine whether the broadband noise acoustic stapedial reflex threshold advantage over pure-tone acoustic reflex thresholds is reduced in individuals with clinically hidden auditory complaints despite normal conventional audiometric thresholds. We use “hidden” in an operational clinical sense to refer to auditory difficulty not detected by the standard 250–8000 Hz audiogram, without implying confirmed cochlear synaptopathy or a specific lesion site. Because vulnerability to synaptic damage increases with aging and prolonged noise exposure [12], we expected that symptomatic ears with age-related cochlear and neural changes might show a reduced BBN advantage despite normal hearing thresholds at conventional frequencies.

## 2. Materials and Methods

### 2.1. Participants

Participants were recruited from UBC and auditory clinics in the Greater Vancouver region in the Hidden Hearing Loss (HHL) study (n = 73 participants, n = 146 ears). The study procedures received ethics approval as part of an HHL project in the UBC Middle Ear Lab. The inclusion criteria of the HHL study were normal hearing thresholds of 25 dB HL or better with no air–bone gap at the conventional audiometric frequencies of 250 to 4000 Hz; normal middle ear function as assessed by wideband acoustic immittance (WAI), presence of robust otoacoustic emission (OAE), and acoustic stapedial reflex; and willingness to provide informed consent. The original dataset included 146 ear-level observations from 73 participants. Thirteen ear-level observations contained one or more missing values in variables required for analysis, including 25 missing ASRT values (out of 438), and 12 missing values for sex.

**Figure 1:**
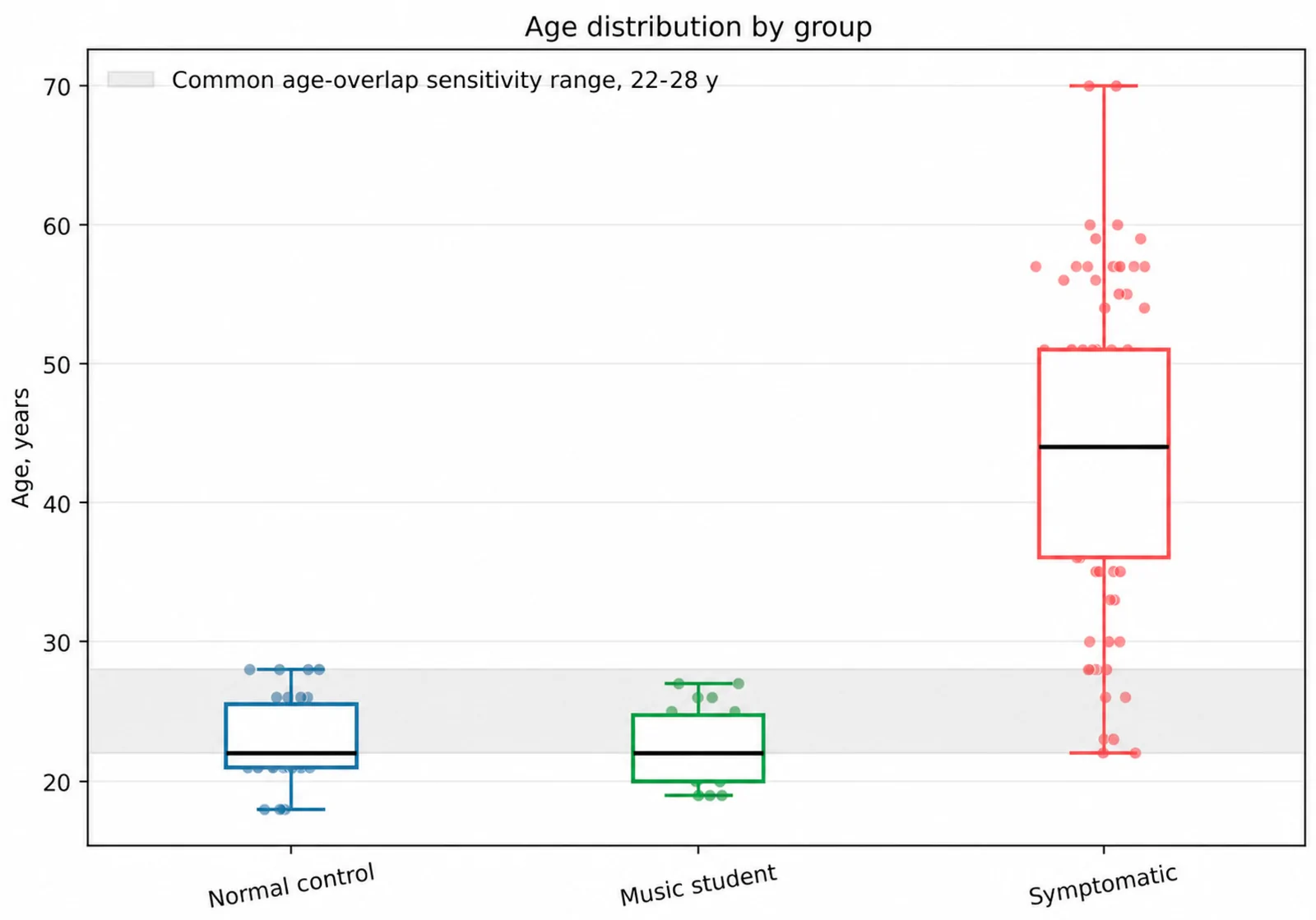
Boxplots show the median and interquartile range. Whiskers extend to 1.5 times the interquartile range, and points represent individual ear-level observations. The shaded region indicates the common age-overlap range used in the age-restricted sensitivity analysis in the Results Section.

These participants were categorized into three groups according to their past noise exposure and clinical symptoms: (1) UBC normal-hearing students (Control students) who served as the control group and self-reported without prolonged musical exposure (n = 15 participants, n = 30 ears, mean age 22.6 yrs with an age range of 18–28); (2) UBC music students (Music students), who were students of the music major with prolonged loud noise exposure (n = 11 participants, n = 22 ears, mean age 22.5 yrs with an age range of 19–27); and (3) symptomatic patients (Symptomatic) who self-reported hearing difficulties understanding speech in noisy environments despite a normal audiogram result (n = 41 participants, n = 81 ears, mean age 44.2 yrs with an age range of 22–70). The symptomatic group was defined operationally as individuals with clinically hidden auditory complaints, meaning individuals who reported difficulty understanding speech in noise, tinnitus, or both, despite normal conventional audiometric thresholds from 250–8000 Hz. All participants had a normal audiogram at conventional frequencies. These participants were referred to the Middle Ear Lab. Figure 1 shows the distribution of age by group in box plots, which present the median age and interquartile range for each group. The shaded region indicates the common age overlap. The symptomatic group was substantially older than the normal control and music student groups, highlighting potential residual confounding by age.

### 2.2. Data Collection

Data were recorded in the Middle Ear Lab from 2017 to 2019, and data extraction and statistical analyses were conducted between 2025 and 2026. All participants first underwent a hearing screening. First, a Welch Allyn otoscope was used to perform otoscopy and assess for occluding cerumen or other abnormalities in the ear canal. Lifetime noise exposure was estimated using the Noise Exposure Structured Interview (NESI). NESI was administered only to the control group and the music students to estimate overall lifetime noise exposure and to investigate the impact of noise on all test protocols used in this study. This technique estimates exposure level (SPL) based on the participant’s self-reported vocal effort during the activity. For this study, raw NESI scores were used to measure participants’ lifetime noise exposure [23]. Differences in NESI scores between the two groups were small and of uncertain sign. The automatic and adaptive Bekesy method (with pulsed tones in 1 dB steps) was used to obtain pure-tone thresholds in the conventional (250–8000 Hz) and extended high-frequency (9–16 kHz) ranges, using HDA-200 circumaural headphones connected to an Otometrics Astera audiometer. The audiometer was calibrated annually by a certified technician.

Middle ear status, as well as OAEs (DPOAE and TEOAE; not analyzed for this study), was measured with an Interacoustics Titan (software suite version 3.4.1) connected to a Windows computer running Interacoustics Otoaccess database software version V.121, before acoustic reflex testing. The system was calibrated before testing participants. Only participants demonstrating a normal wideband absorbance pattern with the presence of the acoustic stapedial reflex, present otoacoustic emissions (at least 3/5 TEOAEs and 4000 Hz DPOAE and TEOAE), and normal 226 Hz tympanometry within established normative ranges were included, thereby minimizing the influence of middle ear pathology on acoustic reflex threshold measurements. Acoustic reflex testing was performed in both ears using automated threshold estimation with a 0.02 mmho criterion change from baseline. Participants’ demographic information, including age, ethnicity, and sex, was collected along with the acoustic reflex threshold.

### 2.3. ASRT Measurements and ASRT Difference Scores

The dependent variable was the acoustic stapedial reflex thresholds (ASRTs, in dB HL) measured with pure-tone stimuli (500 and 2000 Hz) and BBN stimuli. ASRTs were recorded by identifying an admittance change of >0.02 mmho in response to pure-tone stimuli at 500 and 2000 Hz or to BBN stimuli in both ears of all participants [24]. Three types of ASRT difference scores were calculated: the 500 Hz difference (“500Diff”), the 2000 Hz difference (“2000Diff”), and the average difference (“AvgDiff”). The AvgDiff was calculated using Equation (1). Both 500Diff and 2000Diff were calculated using Equation (2).

(1) $$AvgDiff\;\left(dB\right)=(500\;Hz+2000\;Hz\;ASRT)/2-BBN\;ASRT$$

**Equation (1).** Calculation of Average difference (AvgDiff) between the pure tone and the BBN stimuli.

(2) $$500Diff\;\vee \;2000Diff\;\left(dB\right)=500\;Hz\;ASRT\;\vee \;2000\;Hz\;ASRT-BBN\;ASRT$$

**Equation (2).** Calculation of 500Diff or 2000Diff between pure tone and the BBN stimuli.

According to Equations (1) and (2), a positive AvgDiff indicates that the BBN ASRT was lower than the pure-tone ASRT, defining a “BBN advantage”, while a value near zero or a negative value indicates attenuation or loss of the expected BBN advantage. Additional covariates included age (Age=| testdate-birthdate|) and sex (female or male).

### 2.4. Statistical Analysis

ASRT results and group information from the HHL dataset were extracted and filtered between October and November 2025. The data were analyzed between January and April 2026 using descriptive and regression analyses. Descriptive statistics were used to create a demographic table summarizing baseline characteristics of participants’ ears. ASRT values (in dB HL) were summarized with means and standard deviations (SDs) because they are continuous and approximately normally distributed.

Two outcome variables were analyzed: ASRT values and ASRT difference scores. Bayesian multilevel linear regression models were used to estimate group differences as a function of either Reflex Type (three levels: 500 Hz, 2000 Hz, or BBN) or ASRT difference scores (500Diff, 2000Diff, and AvgDiff). Group (three levels), Sex (two levels), and the interaction between Group and Reflex Type were included as categorical predictors. Age was included as a continuous predictor. By-participant and participant-by-ear interaction varying (random) intercepts were included to account for the correlation between ears from the same participant. In the model for the ASRT outcomes, a participant-level varying effect was also included. Weakly regularizing priors were used for all coefficients. Specifically, the intercept prior was specified as Normal (85, 4), the prior for the sex coefficient was Normal (0, 4), and the prior for age was Normal (0, 0.2). Participant-level varying effect parameters were assigned Exponential (1) priors. Marginal posterior means were estimated as distribution medians; 90% uncertainty intervals (UIs) are reported for each median estimate.

To assess whether the group differences were robust to the choice of outcome and statistical specification, a multilevel regression model was fitted using AvgDiff, the average BBN advantage relative to the 500 and 2000 Hz ASRTs, as the primary outcome. The multilevel structure accounted for correlations between ears within the same participant and adjusted group comparisons for age and other specified covariates. This model included categorical predictors of Group, Age, and Sex as covariates, and varying intercepts by Participant. For this model, the intercept, Sex, and Group priors were specified as Normal (0, 4), and the priors for Age and the varying intercepts were the same as for the previous, stimulus-specific model. To evaluate the influence of age imbalance among groups, additional exploratory sensitivity analyses were performed in age-restricted subsets, including the common age-overlap range of 22–28 years and a young-only subgroup of participants aged 30 years or younger. 

Receiver operating characteristic (ROC) analysis was conducted as an exploratory secondary analysis to assess how well BBN advantage measures, calculated as ASRT difference scores, separated symptomatic from non-symptomatic ears in the study sample. Symptomatic ears were coded as the positive class, while normal control and music student ears were combined as the comparison group. The area under the curve (AUC) was used to summarize discrimination. ROC curves were generated for AvgDiff, 500Diff, and 2000Diff, with lower AUC values interpreted as indicating reduced BBN advantage. Thresholds were selected using Youden’s index, and sensitivity and specificity were reported descriptively. Because symptom status was not an independently validated diagnostic reference standard, the current ROC results cannot be interpreted as measures of diagnostic accuracy or clinical validity.

Missing values were modeled using a multiple imputation procedure implemented in the “missRanger” (version 2.6.1) package for R [25]. This package uses a random forest algorithm for imputation [26]. Twenty imputations were computed for each regression model, with the posteriors calculated by pooling across all imputations. Bayesian models were implemented with the “brms” (version 2.23) package for R [27,28]. Posterior was estimated by running three parallel Markov Chain Monte Carlo chains, each with 500 warm-up and 2500 sampling iterations. Convergence of MCMC chains was verified by observing that ‘Rhat’ values for all parameters did not exceed 1.01, and that the effective sample size was at least 450 for all parameters [27,28]. 

## 3. Results

### 3.1. Descriptive Analysis

**Figure 2:**
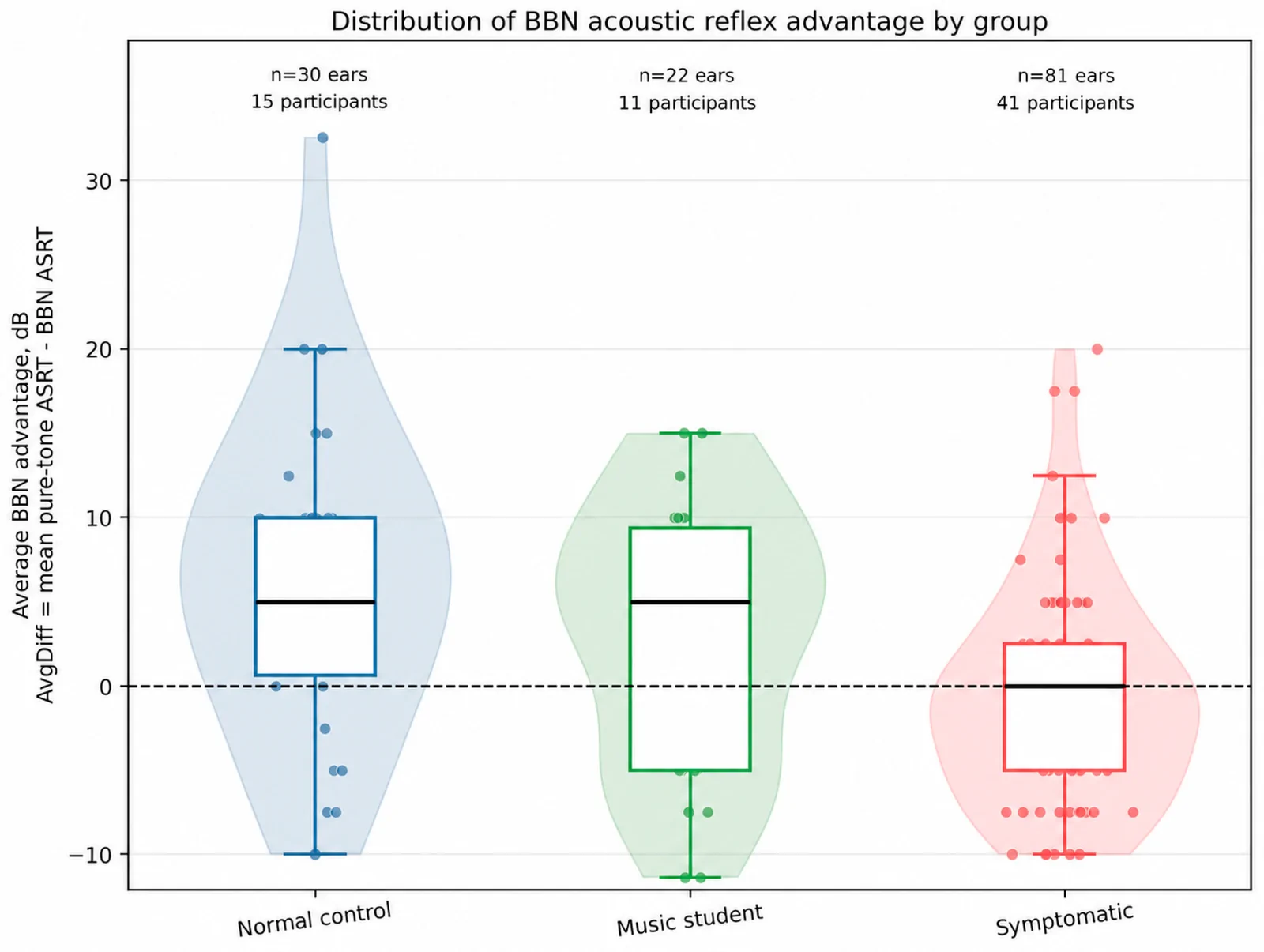
Distribution of BBN acoustic reflex advantage by group. AvgDiff was calculated as the average pure-tone ASRT minus the BBN ASRT. Positive values indicate lower BBN thresholds relative to pure-tone thresholds, consistent with preserved BBN advantage. Values near zero or below zero indicate reduced or absent BBN advantage. Violin plots show the distribution of values, boxplots show the median and interquartile range, whiskers extend to 1.5 times the interquartile range, and points represent individual ears. The dashed horizontal line indicates zero difference.

After removing missing values for ASRTs and Sex, a total of 133 ears from 67 participants were included in the descriptive analysis. Table 1 shows that symptomatic ears have higher thresholds for BBN stimuli than the other groups, but the difference is relatively small for pure-tone stimuli; also note that SD values are larger for BBN than for pure-tone stimuli. Control ears had the largest positive mean AvgDiff; music student ears had intermediate threshold values; and symptomatic ears had the lowest mean AvgDiff (close to zero). On average, control ears preserved the expected BBN advantage; music student ears showed a reduced BBN advantage; and symptomatic ears nearly lost it. Figure 2 shows the distributions of AvgDiff across groups. This figure shows considerable overlap in the range of AvgDiff values across groups, but only the symptomatic groups show a clearly positively skewed distribution of AvgDiff values (Figure 2).

### 3.2. Stimulus-Specific ASRT Analysis

**Figure 3:**
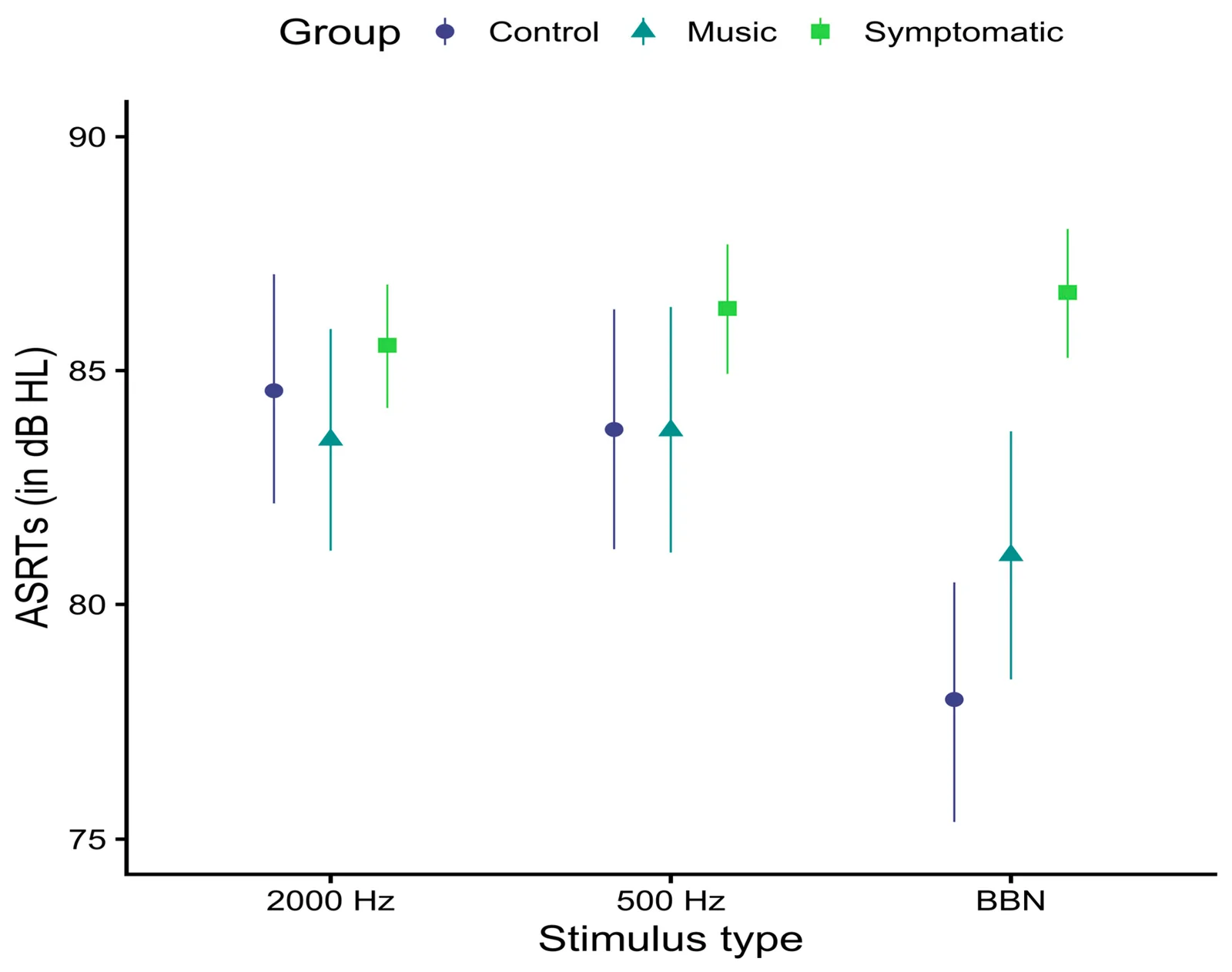
Means of the marginal posteriors for each stimulus type are displayed for each group. Error bars represent 90% uncertainty intervals. Music students are represented by filled circles. Control participants are shown as filled triangles, and symptomatic ears as filled squares.

Figure 3 shows the marginal posterior means with 90% UIs for the Group by Reflex Type interaction. This figure shows that the pattern of ASRTs was similar across the pure-tone stimuli but differed for the BBN stimulus. Specifically, control and music participants have markedly lower thresholds for the BBN than for the pure-tone stimuli, whereas the symptomatic group shows similar thresholds across all stimuli (Figure 3).

To quantify these observations, pairwise contrasts between groups were conducted for each stimulus. The outcomes of these contrasts are reported in Table 2. This table shows that differences between groups for the pure tone stimuli were small to medium in size (relative to an overall SD of 5.85 [5.46, 6.26]) and of uncertain sign. By contrast, differences between the symptomatic and the other groups were relatively large and positive. The effect of Age was small and of uncertain sign (−0.03 [−0.12, 0.06]), while males had overall higher thresholds than females (2.38 [0.59, 4.11]). A considerable amount of variability was accounted for by participant, SD(Interceptprt) = 3.99 [3.28, 4.83] (Table 2). In summary, we observed positive differences with high probability between the symptomatic and other groups only for the BBN stimuli. We also found that males had overall higher thresholds than females.

### 3.3. Group Differences in BBN Advantage

Contrasts were estimated from the regression model posterior, with AvgDiff as the outcome, to assess whether AvgDiff differed across groups, adjusting for age, sex, and participant-level clustering. Table 3 reports the posterior mean differences (and the slope for the Age predictor) and 90% uncertainty intervals for each comparison. The table shows that the difference between symptomatic and control ears was large and negative, with high probability (−6.99, [−10.17, −2.27]). Music student ears also had lower AvgDiff than control ears, but the sign of the difference was less certain (−2.83, [−6.66, 0.98]). AvgDiff was lower for the symptomatic group than for the music student group, but the sign of this difference was uncertain (Table 3). Table 3 also shows that the mean effect of Age was small and of uncertain sign, and that the effect of Sex was consistent with the effect reported for the stimulus-specific ASRT analysis, though its sign was uncertain. Variability due to participant differences, estimated as SD(Interceptparticipant) = 4.92 [3.5, 6.18], was relatively large (Table 3).

### 3.4. Sensitivity Analyses

Because the symptomatic group was substantially older than the normal control and music student groups, an age-restricted sensitivity analysis was performed using the stimulus-specific model. In the common age-overlap subgroup restricted to participants aged 22–28 years, the analysis included 42 ears from 21 participants. Mean age was 24.88 years in normal controls, 24.29 years in music students, and 25.40 years in symptomatic participants. The overall pattern of mean thresholds remained consistent with that of the complete-sample analysis. Symptomatic ears showed higher BBN ASRTs than control ears (8.15 [3.30, 12.80]) and music student ears (2.34 [−2.50, 6.64]); music students had higher ASRT estimates than control participants (−5.80 [−9.57, −1.73]). However, the sign of the difference between symptomatic and music student ears became uncertain in the age-matched sample; by contrast, the sign of the music vs. control difference is clearly negative in the current sample but was uncertain in the complete sample.

A second sensitivity analysis with the age-matched groups was performed with AvgDiff as the outcome. This analysis replicated the reduced BBN advantage for symptomatic vs. control ears (−6.80 [−11.80, −1.87]). A larger BBN advantage was also observed for control than music student ears (5.25 [1.26, 9.31]). These sensitivity analyses supported the direction of the previous findings but had wider uncertainty intervals overall due to the smaller sample size.

### 3.5. Test Performance Analysis

**Figure 4:**
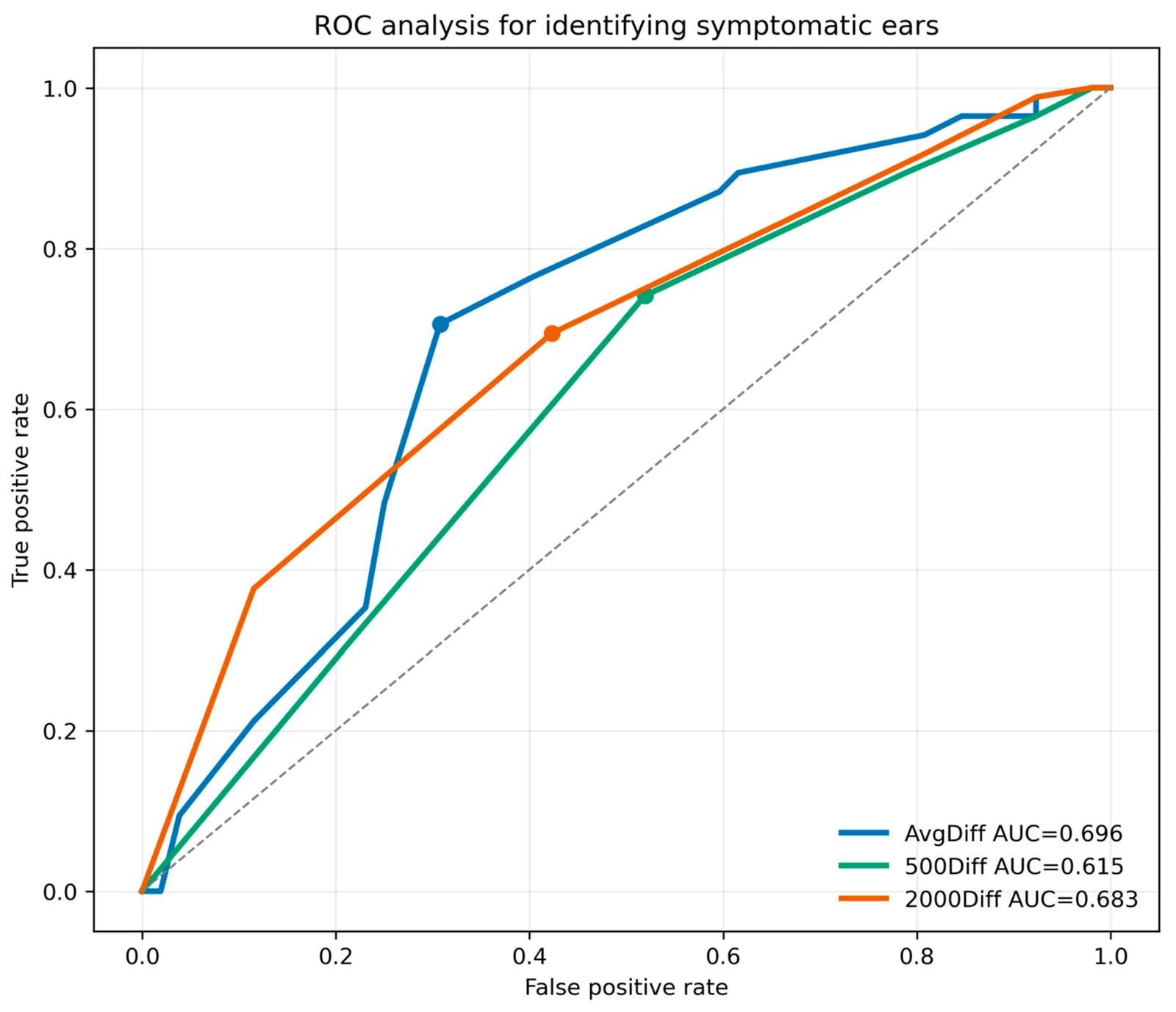
Receiver operating characteristic analysis for identifying symptomatic ears. ROC curves are shown for AvgDiff, 500Diff, and 2000Diff. Lower values of each metric were treated as indicating a greater likelihood of symptomatic status. The diagonal dashed line represents chance-level performance. Dots indicate the Youden-optimal threshold for each metric.

Figure 4 shows the output of an exploratory ROC analysis that was carried out to compare the classification accuracy of the AvgDiff, 500Diff and 2000Diff metrics. AvgDiff showed moderate separation of symptomatic from non-symptomatic ears, with an AUC of 0.696. The Youden-selected cutoff was AvgDiff ≤ 0, yielding sensitivity of 70.6% and specificity of 69.2%. 2000Diff showed slightly lower discrimination than AvgDiff, whereas 500Diff showed the weakest discrimination (Figure 4). These results should be interpreted only descriptively, as symptom status was not determined using an independent diagnostic reference standard.

## 4. Discussion

The present findings show that symptomatic ears consistently had higher BBN ASRTs than control ears, and that music students had intermediate thresholds. We also found that symptomatic ears had a lower AvgDiff than normal control ears, indicating a reduced BBN acoustic reflex advantage in the symptomatic group. Similar findings were observed in stimulus-specific analyses. The Bayesian analysis provided strong directional evidence for the symptomatic-versus-normal comparison, although the wide uncertainty intervals indicate uncertainty about the magnitude of the effect. Comparisons involving music students were directionally consistent with the hypothesized graded pattern, but there was uncertainty about the sign of the difference relative to control ears. The observed pattern is biologically plausible because BBN stimulation recruits a broader cochlear input than pure-tone stimulation, and the BBN advantage may depend on the integrity of distributed cochlear and auditory neural circuits [29,30]. The prior literature suggests that aging and noise exposure can affect auditory nerve function and cochlear synapses, sometimes in ways not fully captured by conventional audiometry [3,12,20]. However, human studies of middle-ear muscle reflex measures as markers of noise-induced cochlear damage remain mixed, and further validation is needed [12,15,21,22]. The symptomatic group was defined by auditory complaints despite normal conventional audiometric thresholds, not by direct evidence of cochlear synaptopathy. Therefore, the reduced BBN advantage in the symptomatic group cannot be attributed specifically to synaptopathy. Symptoms such as difficulty hearing in noise and tinnitus may arise from multiple mechanisms, including subtle cochlear dysfunction, auditory nerve dysfunction, extended high-frequency hearing loss, central auditory processing differences, cognitive or attentional factors, or combinations of these mechanisms [12,15,21,22]. The observed reduction in BBN advantage therefore identifies an association with clinically hidden auditory complaints, but not a definitive mechanism.

### 4.1. Age-Related Sensitivity Analyses

Age imbalance remains an important limitation of this study. The symptomatic group was substantially older than the normal control and music student groups, and age-related cochlear, neural, or central auditory changes may have contributed to the observed reduction in the BBN acoustic reflex advantage [12].

Age-restricted sensitivity analyses showed that the direction of the primary effect persisted across younger and more age-overlapping subgroups. However, these analyses were limited by small sample sizes and wide confidence intervals [31,32]. Thus, while the sensitivity analyses increase confidence that the finding is not solely explained by age, they do not rule out residual confounding. Although age was included as a covariate in the primary Bayesian model, statistical adjustment cannot fully resolve structural confounding when age and group membership are strongly related. Therefore, the symptomatic-versus-normal difference should be interpreted as an association rather than as evidence that symptom status alone caused the reduction in BBN advantage. Future studies should recruit age-matched groups and include objective measures such as speech-in-noise testing, extended high-frequency audiometry, otoacoustic emissions, auditory brainstem response wave I amplitude, envelope-following responses, and detailed lifetime noise-exposure histories.

### 4.2. ROC Analysis

The exploratory ROC analysis suggested that loss or reversal of the BBN advantage may distinguish symptomatic from non-symptomatic ears in this dataset. However, the AUC values were moderate, and symptom status was not validated against a reference standard. Therefore, these findings do not establish diagnostic validity or clinical utility. Future studies should evaluate the BBN advantage against independently defined clinical phenotypes or objective auditory outcomes.

### 4.3. Limitations and Future Studies

Several limitations affected this study. First, the small sample sizes of music students and normal controls led to relatively wide uncertainty intervals, and future studies would benefit from larger samples. Second, this study was cross-sectional; a longitudinal study tracking participants from initial to prolonged exposure over a few years may provide greater statistical power to detect group effects. Third, age imbalance is a major limitation of this study. The symptomatic group was approximately 20 years older on average than the control and music student groups. Future studies with larger sample sizes and age-matching across groups will be necessary to provide stronger evidence of higher BBN ASRTs and reduced BBN advantage in symptomatic ears. Finally, the AUC analysis should be validated with longitudinal and larger-sample data before clinical application. A possible future research question is whether the music student group represents an early-stage or at-risk group for auditory complaints associated with normal audiograms, given the intermediate loss of BBN observed in this study.

## 5. Conclusions

Symptomatic ears showed reduced BBN acoustic stapedial reflex advantage compared with control ears in this exploratory study. The primary finding persisted in participant-level and age-restricted sensitivity analyses, but the study was limited by substantial age imbalance, modest subgroup sizes, and lack of an independent diagnostic reference standard. Therefore, reduced BBN advantage should be interpreted as a promising physiologic observation rather than a validated diagnostic marker. Future age-matched studies incorporating objective auditory measures and detailed noise-exposure histories are needed to determine whether BBN advantage loss reflects subtle diffuse cochlear or auditory neural pathology.

## Figures and Tables

**Table 1:** Participant and ear-level descriptive characteristics by group (adjusted with age and sex).

	Normal Control	Music Student	Symptomatic
No. of ears, n	30	22	85
No. of participants, n	15	11	44
500 Hz ASRT (SD), dB HL	84.1 (5.3)	83.9 (5.0)	86.4 (5.9)
2000 Hz ASRT (SD), dB HL	85.5 (6.0)	83.4 (6.2)	85.5 (5.4)
BBN ASRT (SD), dB HL	78.2 (10.6)	80.7 (9.6)	86.7 (9.2)
AvgDiff, mean (SD), dB HL	6.58 (9.57)	2.95 (7.85)	−0.59 (6.72)

**Table 2:** Group contrasts for ASRTs (in dB HL units) for 500 Hz, 2000 Hz and BBN stimuli.

	Posterior Mean Differences	90% Uncertainty Intervals	Posterior Probability
**500 Hz ASRTs**			
Control—Music students	0.06	[−3.25, 3.39]	Pr(β > 0) = 0.50
Symptomatic—Control	2.69	[−0.71, 6.04]	Pr(β > 0) = 0.90
Symptomatic—Music student	2.76	[−0.60, 6.11]	Pr(β > 0) = 0.90
**2000 Diff ASRTs**			
Control—Music student	0.95	[−1.97, 3.93]	Pr(β > 0) = 0.67
Symptomatic—Control	1.10	[−2.16, 4.37]	Pr(β > 0) = 0.69
Symptomatic -Music student	2.06	[−1.02, 5.07]	Pr(β > 0) = 0.85
**BBN ASRTs**			
Control—Music student	−3.18	[−6.51, 0.17]	Pr(β < 0) = 0.94
Symptomatic—Control	8.73	[5.31, 12.04]	Pr(β > 0) >0.99
Symptomatic—Music student	5.55	[2.17, 8.88]	Pr(β > 0) > 0.99

**Table 3:** Bayesian analysis output for AvgDiff Multilevel linear regression analysis of factors associated with Avg ASRT Differences (adjusted for age and sex).

	Posterior Mean Differences	90% Uncertainty Intervals	Posterior Probability
**Comparison**			
Control—Music students	3.22	−0.14 to 6.62	Pr(β > 0) = 0.94
Symptomatic—Control	−5.89	−9.81 to −2.14	Pr(β < 0) = 0.99
Symptomatic—Music student	−2.66	−6.17 to 1.00	Pr(β < 0) = 0.90
Male vs. Female	−1.67	−4.10 to 0.72	Pr(β < 0) = 0.86
Age, per year	−0.02	−0.14 to 0.09	Pr(β < 0) = 0.65

## Data Availability

We cannot make the data available publicly as it was not included in our approval from the Clinical Research Ethics Board (CREB) at the University of British Columbia.

## References

[1] Wilson B.S., Tucci D.L., O'DOnoghue G.M., Merson M.H., Frankish H. (2019). A Lancet Commission to address the global burden of hearing loss. Lancet.

[2] Liu J., Stohl J., Overath T. (2024). Hidden hearing loss: Fifteen years at a glance. Hear. Res..

[3] Kohrman D.C., Wan G., Cassinotti L., Corfas G. (2020). Hidden Hearing Loss: A Disorder with Multiple Etiologies and Mechanisms. Cold Spring Harb. Perspect. Med..

[4] Valderrama J.T., de la Torre A., McAlpine D. (2022). The hunt for hidden hearing loss in humans: From preclinical studies to effective interventions. Front. Neurosci..

[5] Bramhall N., Beach E.F., Epp B., Le Prell C.G., Lopez-Poveda E.A., Plack C.J., Schaette R., Verhulst S., Canlon B. (2019). The search for noise-induced cochlear synaptopathy in humans: Mission impossible?. Hear. Res..

[6] Kujawa S.G., Liberman M.C. (2015). Synaptopathy in the noise-exposed and aging cochlea: Primary neural degeneration in acquired sensorineural hearing loss. Hear. Res..

[7] Paul B.T., Bruce I.C., Roberts L.E. (2017). Evidence that hidden hearing loss underlies amplitude modulation encoding deficits in individuals with and without tinnitus. Hear. Res..

[8] Kujawa S.G., Liberman M.C. (2009). Adding insult to injury: Cochlear nerve degeneration after “temporary” noise-induced hearing loss. J. Neurosci..

[9] Pienkowski M. (2018). Prolonged Exposure of CBA/Ca Mice to Moderately Loud Noise Can Cause Cochlear Synaptopathy but Not Tinnitus or Hyperacusis as Assessed With the Acoustic Startle Reflex. Trends Hear..

[10] Grinn S.K., Wiseman K.B., Baker J.A., Le Prell C.G. (2017). Hidden Hearing Loss? No Effect of Common Recreational Noise Exposure on Cochlear Nerve Response Amplitude in Humans. Front. Neurosci..

[11] Shehabi A.M., Prendergast G., Guest H., Plack C.J. (2022). The Effect of Lifetime Noise Exposure and Aging on Speech-Perception-in-Noise Ability and Self-Reported Hearing Symptoms: An Online Study. Front. Aging Neurosci..

[12] Shehabi A.M., Prendergast G., Plack C.J. (2022). The Relative and Combined Effects of Noise Exposure and Aging on Auditory Peripheral Neural Deafferentation: A Narrative Review. Front. Aging Neurosci..

[13] Alagramam K.N., Weisz C.J.C., England J.D. (2025). Auditory system, central. Encyclopedia of the Neurological Sciences.

[14] Wojtczak M., Beim J.A., Oxenham A.J. (2017). Weak Middle-Ear-Muscle Reflex in Humans with Noise-Induced Tinnitus and Normal Hearing May Reflect Cochlear Synaptopathy. eNeuro.

[15] Trevino M., Zang A., Lobarinas E. (2023). The middle ear muscle reflex: Current and future role in assessing noise-induced cochlear damage. J. Acoust. Soc. Am..

[16] Hall J.W. (1978). Predicting hearing level from the acoustic reflex. A comparison of three methods. Arch. Otolaryngol..

[17] Interacoustics Acoustic Reflex Testing: A Complete Guide 2026. https://www.interacoustics.com/academy/tympanometry-training/acoustic-reflexes/acoustic-reflex-testing.

[18] Wallin A., Mendez-Kurtz L., Silman S. (1986). Prediction of hearing loss from acoustic-reflex thresholds in the older adult population. Ear Hear..

[19] Silman S., Silverman C.A., Showers T., Gelfand S.A. (1984). Effect of age on prediction of hearing loss with the bivariate-plotting procedure. J. Speech Lang. Hear. Res..

[20] Shenoy S., Bhatt K., Yazdani Y., Rahimian H., Djalilian H.R., Abouzari M. (2025). A Systematic Review: State of the Science on Diagnostics of Hidden Hearing Loss. Diagnostics.

[21] Valero M.D., Hancock K.E., Liberman M.C. (2016). The middle ear muscle reflex in the diagnosis of cochlear neuropathy. Hear. Res..

[22] Guest H., Munro K.J., Plack C.J. (2019). Acoustic middle-ear-muscle-reflex thresholds in humans with normal audiograms: No relations to tinnitus, speech perception in noise, or noise exposure. Neuroscience.

[23] Guest H., Dewey R.S., Plack C.J., Couth S., Prendergast G., Bakay W., Hall D.A. (2018). The Noise Exposure Structured Interview (NESI): An Instrument for the Comprehensive Estimation of Lifetime Noise Exposure. Trends Hear..

[24] Hunter L.L., Shahnaz N. (2013). Acoustic Immittance Measures: Basic and Advanced Practice.

[25] Rubin D.B. (1986). Statistical matching using file concatenation with adjusted weights and multiple imputations. J. Bus. Econ. Stat..

[26] Wright M.N., Ziegler A. (2017). Ranger: A fast implementation of random forests for high dimensional data in C++ and R. J. Stat. Softw..

[27] Bürkner P.-C. (2017). Advanced Bayesian multilevel modeling with the R package brms. arXiv.

[28] R Core Team (2026). R: A Language and Environment for Statistical Computing. https://www.R-project.org/.

[29] Versteegh C.P., van der Heijden M. (2012). Basilar membrane responses to tones and tone complexes: Nonlinear effects of stimulus intensity. J. Assoc. Res. Otolaryngol..

[30] Eggermont J.J., Levin K.H., Chauvel P. (2019). Chapter 29—Cochlea and Auditory Nerve, in Handbook of Clinical Neurology.

[31] Faber J., Fonseca L.M. (2014). How sample size influences research outcomes. Dent. Press J. Orthod..

[32] Hespanhol L., Vallio C.S., Costa L.M., Saragiotto B.T. (2019). Understanding and interpreting confidence and credible intervals around effect estimates. Braz. J. Phys. Ther..

